# Muscle tendon elongation with bovine pericardium (Tutopatch®) in patients with Graves’ orbitopathy; a prospective, observational, multicentre study

**DOI:** 10.1111/aos.17545

**Published:** 2025-07-08

**Authors:** H. M. Jellema, M. Oeverhaus, A. Eckstein, I. Mombaerts, R. J. H. M. Kloos, D. T. Hartong, P. Nieuwkerk, P. Saeed

**Affiliations:** ^1^ Department of Ophthalmology, Orbital Center, Amsterdam UMC University of Amsterdam Amsterdam The Netherlands; ^2^ Department of Ophthalmology University Duisburg Essen Essen Germany; ^3^ Department of Ophthalmology University Hospitals Leuven Leuven Belgium; ^4^ Department of Neurosciences, Faculty of Medicine Catholic University Leuven Leuven Belgium; ^5^ Department of Medical Psychology, Amsterdam UMC University of Amsterdam Amsterdam The Netherlands

**Keywords:** bovine pericardium, diplopia, extraocular muscle tendon elongation, Graves’ orbitopathy, HADS, Tutopatch

## Abstract

**Purpose:**

Patients with severe ocular motility restriction and/or large angle strabismus due to Graves' orbitopathy (GO) cannot be adequately addressed with conventional strabismus surgery using large muscle recessions. Muscle tendon elongation surgery, using a spacer of bovine pericardium (Tutopatch®) has emerged as a valuable alternative. The purpose of this study was to assess the long‐term outcome of muscle elongation surgery in patients with GO.

**Methods:**

A prospective observational cohort study in three University centres was conducted. All GO patients requiring strabismus surgery with elongation material of one or more rectus muscles were included. One week preoperatively and 3 and 6 months postoperatively, ophthalmic and orthoptic examinations were performed.

**Results:**

Thirty‐five patients were enrolled in the study. The mean length of spacer used was 8.2 mm (range 4–13 mm). Tendon elongation was performed on 15 inferior rectus muscles, 33 medial rectus muscles and 9 superior rectus muscles. The field of BSV improved from 0 points [IQR 0–0] preoperatively to 80 points [IQR 6–92] postoperatively (*p* < 0.001). QoL for functional questions changed from 47 points [IQR 31–63] preoperatively to 78 points [IQR 73–100] postoperatively (*p* = <0.001). HADS level of anxiety and depression remained below the cut‐off score of 15 points (preoperative 11.5 and postoperative 5.7 points).

**Conclusions:**

Using the muscle tendon elongation technique with Tutopatch® in GO patients with severely restricted strabismus, 3 months after surgery a stable large field of BSV can be achieved with associated improvement of the QoL. It is a safe and effective alternative to large muscle recessions for restricted muscle.

## 1 INTRODUCTION

Graves' orbitopathy (GO) is characterized by eyelid retraction, proptosis, chemosis and swelling of the rectus muscles. Patients experience pain with eye movements, foreign body sensations and double vision. Treatment of GO ranges from drug therapy to various surgical treatments (Bartalena et al., 2021). In patients with thyroid eye disease, the 4‐year cumulative incidence for strabismus is 10% and the cumulative incidence of surgical interventions is 8% for strabismus surgery (Boulakh et al., 2022). Due to the long‐term course of the condition, quality of life (QoL) can be severely impaired, both in appearance and function (Terwee et al., 2002). In addition, hyperthyroidism plays an important negative role in psychiatric morbidity (Bové et al., 2014; Bunevicius et al., 2005; Kahaly et al., 2005) and patients with Graves' disease experience depression and anxiety, for which the Hospital Anxiety and Depression Scale (HADS) questionnaire can be used as a screening tool to detect this. A study by Kahaly et al. (2005) found that a higher number of stressful events was correlated with a higher degree of anxiety and depression. Almost half of the patients with GO complain of restrictions in their daily activities and impaired self‐perception. Especially, patients with diplopia are on sick leave for longer times and are more likely to be disabled (Ponto et al., 2009). Since its evaluation, the GO‐QoL is a widely used questionnaire showing severe negative QoL in GO patients (Terwee et al., 2002), which significantly improves after treatment (Jellema et al., 2014, 2017; Terwee et al., 2001). In patients with GO, treatment for strabismus is challenging, especially in severely affected patients and after decompression. Prior orbital decompression is associated with more strabismus surgeries per patient, with a trend towards a lower success rate for strabismus surgery (Lee et al., 2019). Conventional recession surgery of the affected rectus muscle may be insufficient in a large‐angle strabismus and may result in weakening of the action of the muscle. To avoid reduction of torque, a technique using extension material such as bovine pericardium has been introduced to maintain the arc of contact of the muscle to the globe. This was first described in 1981 using fascia lata (FL) (Salvi et al., 1981), followed by Goretex (Langmann et al., 2006) and later bovine pericardium (Esser et al., 2011) or donor sclera (Thorisdottir et al., 2019). Esser et al. (2011) described a retrospective series of 10 patients with GO in which the inferior rectus was lengthened with bovine pericardium and found a similar dose–response as with conventional inferior rectus surgery. In 2018, Oeverhaus, Fischer, Hirche, et al. (2018); Oeverhaus, Fischer, Schlüter, et al. (2018) presented a retrospective series of 60 patients with esotropia after decompression surgery in which the rectus medialis was unilaterally or bilaterally elongated using Tutopatch®. Lower dose–response was achieved compared to conventional surgery, and a step‐by‐step approach was recommended. In contrast, Prinz et al. (2022) wrote that bovine pericardium had a higher dose–response than FL in patients after inferior rectus recession because FL was a thicker and less flexible material.

Commonly used success criteria are the required amount of prism (Esser et al., 2011) size of field of binocular single vision (BSV) (Prinz et al., 2022) and stability over time (Wipf et al., 2018). However, the impact of this type of surgery on QoL is lacking.

The purpose of this study was to prospectively investigate the results of muscle tendon lengthening in patients with GO with severe horizontal or vertical restrictive strabismus. Effects on eye position, ductions and cyclotorsion, as well as the field of BSV, QoL and anxiety and depression will be analysed.

## 2 MATERIALS AND METHODS

We conducted a prospective observational cohort study in three tertiary referral centres specialised in orbital surgery in Germany (Essen), Belgium (Leuven) and the Netherlands (Amsterdam). The study was conducted according to the principles of the Declaration of Helsinki (seventh edition, October 2008, Seoul) and in accordance with the Medical Research Involving Human Subjects Act (WMO). Each centre obtained Institutional Review Board or Ethics Committee approval and the Amsterdam centre served as the primary study centre. From January 2018 until December 2020, all patients diagnosed with Graves' Orbitopathy and scheduled for a primary strabismus surgery with bovine pericardium (Tutopatch) on one or more rectus muscles were potentially eligible to participate in the study. Tutopatch (Tutogen Medical GmbH, Neuenkirchen am Brand, Germany) is a biological tissue graft made from a preserved pericardial membrane derived from a bovine (cow) tissue.

Further inclusion criteria were as follows: clinically and biochemically stable, inactive GO, orbital decompression at least 3 months prior to strabismus surgery, stable orthoptic examination of at least 3 months meaning <5 prism dioptres (PD) change in primary position and <8° change in duction (Haggerty et al., 2005; Prummel et al., 2004). Exclusion criteria were as follows: suppression, pre‐existing diplopia not related to the GO, vision less than 0.7 Logmar (0.2 Snellen) and simultaneously conventional recession of another muscle acting in the same direction (horizontal or vertical) as the Tutopatch® muscle.

Data were collected regarding gender, race, marital status, age at surgery, date of first diagnosis of GO, prior treatment for Graves thyroid disease (GTD) and GO, current smoker and diplopia complaints One week preoperatively and 3 and 6 months postoperatively, an ophthalmic and orthoptic exam was performed. If additional strabismus surgery was obligatory, this was scheduled after the 6‐month postoperative visit.

Examination included: prism covertest in primary position at near (30 cm) and distance (5 m) with fixation of the least limited eye at a spotlight wearing optimal correction (without prisms) and without abnormal head posture; ductions measured with the modified perimeter (Mourits et al., 1994) or Goldmann perimeter (Gerling et al., 1997) in four directions of gaze; abduction, adduction, elevation and depression; field of BSV measured with A: the Goldmann perimeter using a III4e light target (Woodruff et al., 1987) and scored as suggested by Sullivan et al. (1992) at a score sheet of 0–100% and B: the Harmswand or Maddox tangent screen on 2.5 m and calculated with the modified diplopia/BSV score sheet (Holmes et al., 2005) without glasses; cyclotorsion (voluntary) measured with the Harmswand or the Maddox tangent screen on 2.5 m with help of the cycloforometer of Franceschetti in primary position and 25° up‐ and downgaze (if the elevation and depression exceeded the 25°), where the minus number means an incyclodeviation. Measurements were performed by orthoptists experienced to examine patients with GO. They were familiar with the tests and received no extra training.

### 2.1 Quality of life and anxiety and depression

Patients were asked to complete the GO‐Qol and the hospital anxiety and depression questionnaire (HADS) after each consultation. Both questionnaires were provided in the Dutch, German or English language version, depending on the patients' language preference. All GO‐QoL questions were scored as ‘severely limited’ (one point), ‘a little limited’ (two points) or ‘not limited at all’ (three points). The questions 1–8 for visual functioning (VF) and questions 9–16 for appearance (AP) were summed to produce two raw scores from 8 to 24 points, and then transformed to two total scores from 0 to 100 by the following formula: total score = [(raw score − 8)/16 × 100]. In both cases, higher total scores indicate better QoL. For questions 1 and 2, the answers ‘no drivers' licence’ or ‘never learned to ride a bike’ were scored as a missing value. When there were missing values, total scores were calculated for the remaining completed items. The transformation was then adjusted to: total score = [(raw score − *)/(2 × *) × 100] where * is the number of completed items (Terwee et al., 1998).

The HADS questionnaire (Zigmond & Snaith, 1983) consists of 14 questions, with all odd‐numbered questions relating to anxiety and all even‐numbered questions relating to depression. Each question has four responses ranging from none (0) to extreme (3), culminating in a maximum score of 21 points per subscale. The original authors suggested a cut‐off score of 7/8 per subscale for detecting clinically relevant levels of depression or anxiety. The total HADS score, that is, the combined score for depression and anxiety, has the highest positive predictive value to detect clinically relevant levels of psychological distress (Spinhoven et al., 1997). Therefore, we use the total HADS score in the analyses of the present study. A cut‐off score of 15 was suggested by several authors (Ibbotson et al., 1994; Kugaya et al., 2000) and resulted in 80% sensitivity, 76% specificity and a positive predictive value of 41%. Patients received no planned psychosocial care during their treatment for GO.

### 2.2 Surgical procedure

The surgical plan for the first surgery was determined together by the surgeon and the orthoptist. Each experienced centre was free to calculate their own dose effect response upon previous experiences. In general, all centres used 1.3–1.5 degree/mm for medial and superior rectus recession and 2.0 degree mm for inferior recession. If a patient was decompressed, the team in Essen used a lower dose effect response for medial rectus recession (1.0°/mm) since here medial decompression was performed endoscopically, which caused marked prolapse of the medial rectus muscle into the ethmoidal cell (Oeverhaus, Fischer, Hirche, et al., 2018; Oeverhaus, Fischer, Schlüter, et al., 2018). In difficult cases, the other sites could be consulted. In the case of a second procedure, consultation with another site took place via a case report form. All test results were given, and the recommendation for surgery included the suggested muscle(s) to be operated on, including the amount of millimetres. If the primary site did not agree, discussion followed until consensus was reached.

Tutopatch® on the medial rectus muscle was used if 6 mm or more recession was needed. If necessary, an amnion membrane was used to cover a conjunctival defect. Tutopatch® on the inferior rectus or superior rectus muscle was used if 6 mm or more recession was planned.

The Tutopatch® was inserted between the rectus muscle and the sclera. Using vicryl 6/0 sutures (Vicryl TM, Ethicon), one end of the Tutopatch® was sutured to the surgically detached muscle tendon, and the other end of the Tutopatch® was sutured to the sclera at the original muscle insertion, or 3 mm behind the muscle insertion in the case of medial rectus surgery for aesthetic reasons.

**FIGURE 1:**
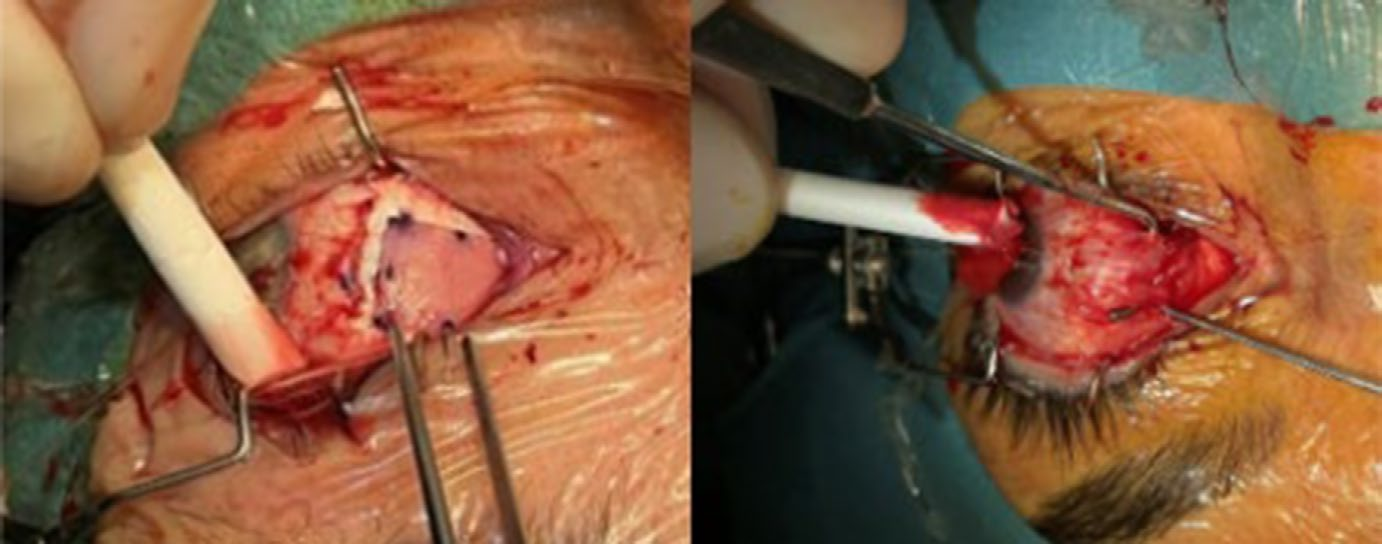
Strabismus surgery: Tutopatch elongation on left medial rectus with 3 mm recession (left) and inspection of Tutopatch 6 months after surgery (right).

The operation was performed by surgeons (AE, IM, RK and PS) experienced in the surgical treatment of GO strabismus and Tutopatch®. Surgery was done under retrobulbar or general anaesthesia (Figure 1). Additional strabismus surgery was scheduled after the 6‐month postoperative visit if required.

### 2.3 Statistical analysis

To answer the research question on the effect of the operation on the field of BSV, we used the results of a similar study (Jellema et al., 2017). This study showed a mean difference of 53 in the field of BSV before and 3 months after surgery, with an associated standard deviation (SD) of 23. Using this data, calculation showed that a sample size of 27 patients will have 80% power to accept or reject the null hypothesis (assuming a single group *t*‐test with a 0.05 two‐sided significant level). To allow for 10% follow‐up, the aim was to include a total of 30 (27 + 2.7) patients.

Statistical analysis was performed with the software package IMB SPSS Statistics (Version 28.0 Corp. in Armonk, New York). Both categorical and continuous data were analysed quantitatively with the appropriate statistical tests. Changes in the field of BSV, QoL scores and degree of strabismus between baseline and 3 and 6 months after treatment were assessed statistically using a paired samples *t*‐test or Wilcoxon signed‐rank test. To detect differences between patient subgroups, the independent sample *t*‐test or Mann–Whitney test was used. Correlations between the field of BSV and QoL pre‐ and postoperatively were analysed with the Spearman rank correlation test. Changes in the HADS scores were analysed with the Wilcoxon rank test, and effect sizes will be calculated (*r* = *z*/√*N*). The association between gender, marital status, age at operation and preoperative HADS scores was investigated with linear regression analysis. Chi‐square was used to investigate differences in total HADS scores ≥15 points (yes/no) by gender, marital status and Gorman score. Linear regression was used to investigate the association between the total HADS score ≥15 points with the onset of GO in months.

## 3 RESULTS

A total of 35 patients were included, 8 patients from Essen, 5 from Leuven and 22 from Amsterdam. Of the cohort, 15 were male and 20 female with a mean age of 60 years (range 38–83 years, see Table 1). In 45% of the patients (31 muscles), Tutopatch® was inserted on the medial rectus muscle for esotropia. The mean lengthening (including 3 mm recession) of the rectus medialis was 8.6 mm (range: 6–13 mm). The preoperative and postoperative angles of deviation are outlined in Table 2. The dose–response was 2.48 ± 0.69 Δ/mm (Table 3) and did not change 6 months after surgery (*p* = 0.438, paired *t*‐test). Abduction increased (preoperatively 19.8 ± 8.5°, postoperatively 32.5 ± 9.6°, *p* = <0.001) and adduction decreased (preoperatively 39.6 ± 5.7°, postoperatively 33.3 ± 9.8°, *p* = 0.002).

**TABLE 1:** Patient characteristics.

	*n* (%) or [range]
Gender	
Male	15 (43%)
Female	20 (57%)
Age	60 [38–83 year]
Time since GO Diagnoses	25 months [4–96]
Prior GTD treatment^a^	
I 131	6
Thyroidectomy	4
Anti‐thyroid drugs	24
None	4
Prior GO treatment^a^	
None	0
Lubricants	20
Selenium	7
Steroids (oral/iv)	10
Other immunosuppressive treatment	8
Radiotherapy	8
Decompression	21
Smoker status (or stopped < 1 year)	
Yes	11
No	24
Unknown	0

Abbreviations: GO, Graves' orbitopathy; GTD, Graves thyroid disease.

^a^ More therapies per patients possible.

**TABLE 2:** Eye position and ductions of patients undergoing medial rectus elongation surgery (*n* = 16).

	Preoperative	3 months postoperative	*p‐*value	6 month postoperative	*p‐*value
	**PD ± SD**			**PD ± SD**	
Horizontal deviation					
Near	42.9 ± 14.3	3.23 ± 8.7	*<0.001*	−2.1 ± 8.3	*0.005*
Distance	45.4 ± 8.7	4.6 ± 8.6	*<0.001*	1.8 ± 8.0	0.341
	**Degrees ± SD**			**Degrees ± SD**	
Abduction	19.8 ± 8.5	32.5 ± 9.6	*<0.001*	34.5 ± 7.4	0.012
Adduction	39.6 ± 5.7	33.3 ± 9.8	*0.002*.	34.3 ± 7.2	0.160

*Note*: italics = level of significance (*p* ≤ 0.05).

Abbreviations: MR, medial rectus muscle; PD, prism dioptres; SD, standard deviation.

**TABLE 3:** Dose effect response for squintangle and cyclotorsion.

Dose effect response squint angle	3 months postoperative	6 months postoperative	*p‐*value
	PD ± SD [range]	PD ± SD [range]	
Medial rectus muscle *n* = 16	2.48 ± 0.69 [1.28–3.60]	2.55 ± 0.74 [1.05–3.92]	0.438
Inferior rectus muscle *n* = 11	3.47 ± 1.11 [1.64–5.33]	4.01 ± 1.40 [1.55–5.56]	0.389
Superior rectus muscle *n* = 6	2.50 ± 0.63 [2.00–3.33]	2.88 ± 0.52 [2.29–3.50]	0.172

Dose effect response cyclotorsion	3 months postoperative	6 months postoperative	*p‐*value
	Degree ± SD [range]	Degree ± SD [range]	
Inferior rectus muscle *n* = 11			
Primary position	+ 0.86 ± 0.26 [0. 44–1.31]	+ 0.99 ± 0.28 [0.60–1.4]	0.258
Downgaze	+ 0.71 ± 0.55 [−0.56–1.43]	+ 0.87 ± 0.30 [0.33–1.23]	0.110
Superior rectus muscle *n* = 6			
Primary position	−0.22 ± 0.31 [−0.69–0.16]	−0.24 ± 0.35 [−0.80–0.10]	0.842
Downgaze	−0.68 ± 0.68 [−1.54 – 0.29]	−0.21 ± 0.45 [−1.00–0.25]	0.386

*Note*: Cyclotorsion: − = incyclotorsion; + = excyclotorsion. Patients with inferior + superior rectus surgery other eye were excluded from this analysis (*n* = 2). Dose effect response was calculated for distance fixation.

Abbreviations: PD, prism dioptres; SD, standard deviation.

Fifty‐four per cent of the patients underwent surgery for a vertical deviation, with a total of 14 inferior rectus muscles and 9 superior rectus muscles. In the latter group, three patients underwent asymmetrical inferior rectus recession and in two patients additional medial rectus surgery was performed (1 Tutopatch® and 1 recession). One inferior rectus muscle detached from the globe due to extreme tightness and could not be reattached. Two patients were operated on the superior rectus and inferior rectus simultaneously using Tutopatch. The mean lengthening of the inferior rectus was 8.0 mm (range 4–11 mm). The dose–response was 3.47 ± 1.11 Δ/mm (Table 3) and did not change 6 months after surgery (*p* = 0.389). The mean lengthening of the superior rectus was 7.4 mm (range 6–10 mm). The dose–response was 2.50 ± 0.63 Δ/mm and remained unchanged 6 months after surgery (*p* = 0.172). Tables 4 and 5 outline the preoperative and postoperative angles of deviations and ductions of vertical elongation surgery. Only the elevation after lengthening of the inferior rectus differs statistically 6 months postoperatively from the value 3 months postoperatively (22.5 ± 8.3 vs. 20.3 ± 10.4). All other measurements show no difference. Overall, no overcorrections were found.

**TABLE 4:** Eye position and ductions of patients undergoing inferior rectus elongation surgery (*n* = 11).

	Preoperative	3 months postoperative	*p‐*value	6 month postoperative	*p‐*value
	**PD ± SD**			**PD ± SD**	
Vertical deviation					
Near	38.6 ± 18.6	11.6 ± 14.8	*<0.001*	7.9 ± 11.9	0.122
Distance	38.4 ± 17.1	11.8 ± 14.2	*<0.001*	9.7 ± 10.4	0.729
	**Degrees ± SD**			**Degrees ± SD**	
Cyclotorsion					
Primary position	+9.5 ± 3.8	+1.3 ± 3.1	*<0.001*	+0.4 ± 2.1	0.589
Downgaze	+8.0 ± 4.9	+2.0 ± 6.3	*0.002*	+0.2 ± 2.2	0.099
Duction					
Elevation	14.9 ± 12.2	22.5 ± 8.3	*0.021*	20.3 ± 10.4	*0.024*
Depression	57.7 ± 5.3	53.1 ± 6.3	*0.017*	52.3 ± 6.4	0.900

*Note*: Cyclotorsion: + = excyclotorsion; − = incyclotorsion; Patients with combined surgery on the superior rectus of the other eye were excluded. italics = level of significance (*p* ≤ 0.05).

Abbreviations: PD, prism dioptres; SD, standard deviation; SR, superior rectus muscle.

**TABLE 5:** Eye position and ductions of patients undergoing superior elongation surgery (*n* = 6).

	Preoperative	3 months postoperative	*p‐*value	6 month postoperative	*p‐*value
	**PD ± SD**			**PD ± SD**	
Vertical deviation					
Near	22.2 ± 8.4	2.3 ± 3.9	*<0.001*	1.5 ± 3.7	0.341
Distance	21.0 ± 8.2	3.2 ± 6.8	*<0.001*	0.3 ± 2.6	0.197
	**Degrees ± SD**			**Degrees ± SD**	
Cyclotorsion					
Primary position	+2.0 ± 2.2	+1.8 ± 2.7	*0.006*	−0,5 ± 0.8	0.058
Downgaze	+2.3 ± 5.0	+0.3 ± 3.1	*0.026*	−1.0 ± 3.1	0.379
Duction					
Elevation	22.8 ± 7.7	17.5 ± 7.1	*0.006*	19.8 ± 7.2	0.116
Depression	55.7 ± 2.9	56.8 ± 2.6	*0.017*	58.2 ± 1.9	0.475

*Note*: Cyclotorsion: + = excyclotorsion; − = incyclotorsion; Patients with combined surgery on the inferior rectus of the other eye were excluded. italics = level of significance (*p* ≤ 0.05).

Abbreviations: PD, prism dioptres; SD, standard deviation; SR, superior rectus muscle.

The mean excyclotorsion decreased, particularly in the inferior rectus group (preoperatively +9.5 ± 3.8° vs postoperatively +1.3 ± 3.1°). Table 3 lists the dose–response of the cyclotorsion in primary gaze and downgaze, for both the inferior and superior rectus muscle elongations.

**FIGURE 2:**
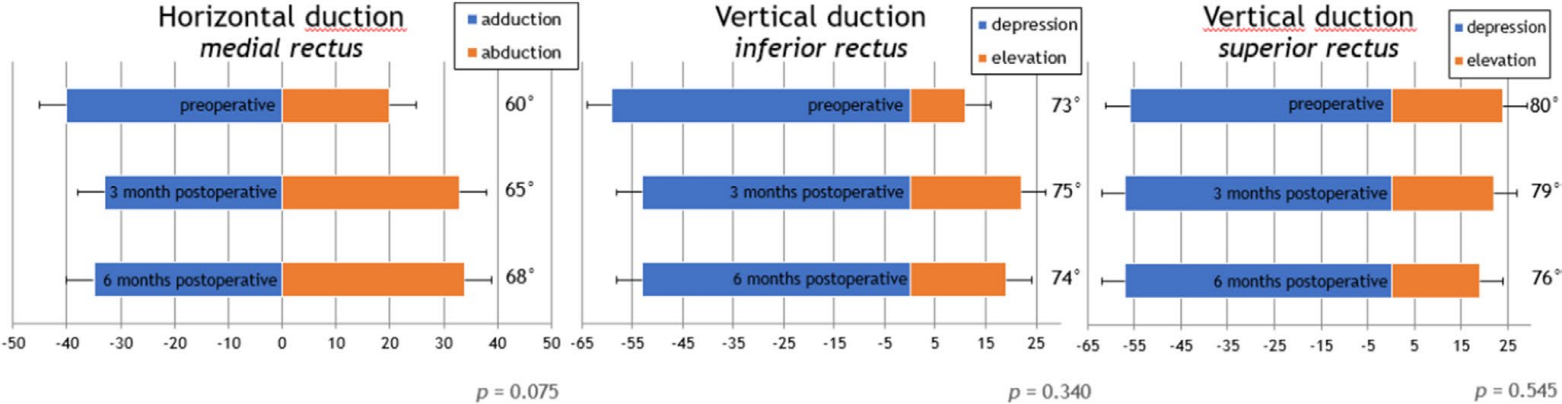
The overall duction range for medial rectus (left), inferior rectus (middle) and superior rectus (right) elongation measured preoperative and 3 and 6 months postoperative.

Ductions in the opposite direction of the elongated muscle increased and ductions in the direction of the operated muscle decreased (Tables 2, 4 and 5). The overall duction range remained stable (inferior rectus elongation *p* = 0.340 and superior rectus elongation *p* = 0.545) and improved slightly in the rectus medialis elongation group (*p* = 0.075; Figure 2).

Eleven patients required additional strabismus surgery for the reason of persisting diplopia. In the case of surgery for a residual angle of deviation in the same direction of gaze (*n* = 8), resection of the lateral rectus muscle (4 muscles), recession of the superior oblique muscle (1 muscle), recession of the superior rectus muscle (4 muscles) and resection of the superior rectus muscle (2 muscles) was performed. The three patients operated on for another type of strabismus received an asymmetrical inferior rectus recession after elongation surgery for the horizontal deviation.

### 3.1 Field of BSV

The field of BSV measured with the Harmswand changed from 0 points (IQR 0–0) to 80 points (IQR 6–92) postoperative (*p* < 0.001 Wilcoxon signed‐rank test) which did not change 6 months postoperative elongation surgery (*p* = 0.913). Goldman values increased from 0 (IQR 0–0) to 55 (IQR 2–78) points postoperatively (*p* < 0.001) and did not change 6 months after surgery (*p* = 0.178). In the cases of additional surgery, the field of BSV with the Harmswand improved from 0 (IQR 0–42) to 8 (IQR 0–67) points 3 months postoperatively (*n* = 5; *p* = 0.317) and increased to 52 (IQR 9–88) points 6 months postoperatively (*n* = 6; *p* = 0.180). Goldman values were similar and changed from preoperative 6 (IQR 0–45) to 39 (IQR 11–65) points 3 months postoperatively (n = 5; *p* = 0.655) and 62 (IQR 9–88) 6 months postoperatively (*n* = 5; *p* = 0.655).

No statistically significant correlation was found when comparing the preoperative score of the field of BSV with the preoperative functional GO‐QoL (*r* = 0.317; *p* = 0.131 Spearman's Rho). However, postoperatively, a moderate correlation was found between the field of BSV and the functional GO‐QoL (*r* = 0.687; *p* = < 0.001).

### 3.2 GO‐QoL and HADS

**FIGURE 3:**
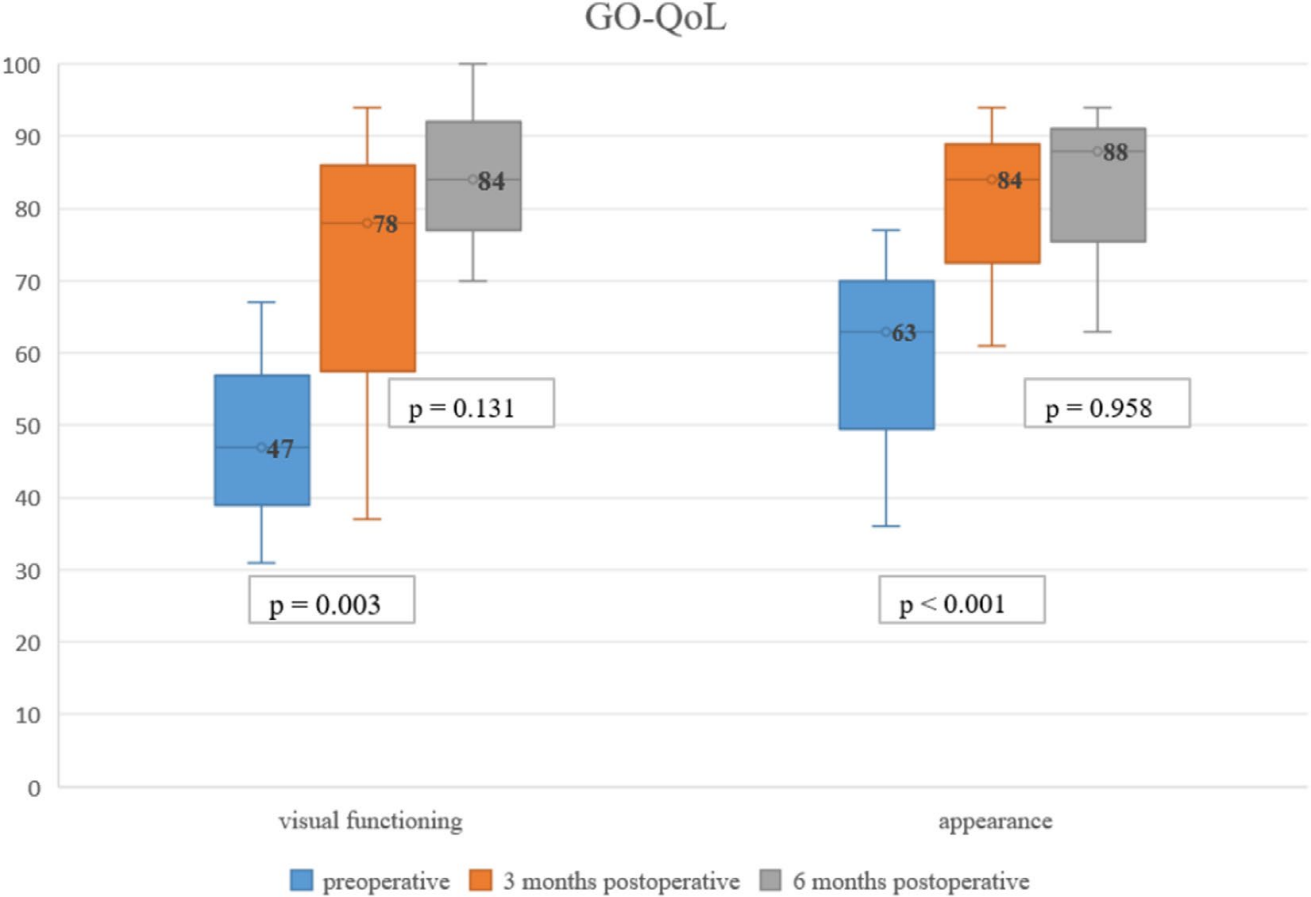
GO‐QoL for visual functioning (left) and appearance measured preoperative and 3 and 6 months postoperative.

GO‐QoL scores increased after elongation surgery (visual functioning *p* = 0.003 and appearance *p* < 0.001) and showed no change 6 months after surgery (visual functioning *p* = 0.131 and appearance *p* = 0.958; Figure 3).

**FIGURE 4:**
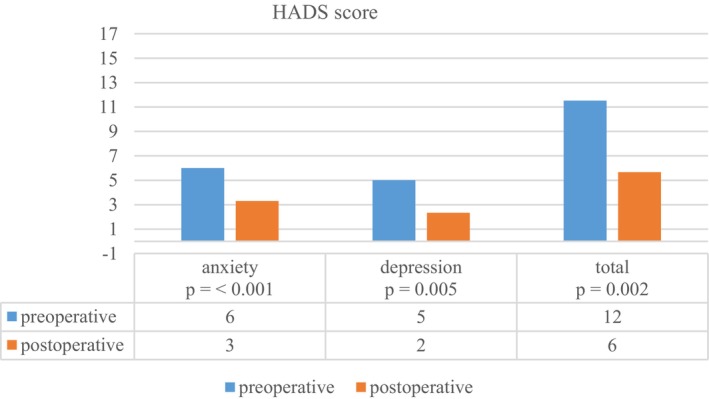
Hospital Anxiety and Depression scale (HADS) questionnaire scores preoperative and 6 months postoperative.

The mean total HADS score decreased from 11.5 points before surgery to 5.7 points after surgery, both below the cut‐off score of 15 points (effect size *r* = 0.627; *p* = 0.002 Wilcoxon rank test; effect size HADS anxiety *r* = 0.675 and HADS depression *r* = 0.564; Figure 4). Neither gender (*p* = 0.931 Chi‐square test), marital status (*p* = 0.931), age at operation (*p* = 0.972), nor preoperative deviation (horizontal *p* = 0.518 and vertical *p* = 0.587) were significantly associated with the preoperative HADS score.

## 4 DISCUSSION

This is the first prospective study analysing the muscle tendon elongation with Tutopatch® in GO patients with severe restricted strabismus. It shows significant improvement of the deviation without worsening of ductions or resulting in overcorrection.

### 4.1 Study cohort

The included patients with severe cases of restrictive strabismus do not reflect a general GO study population. Unsurprisingly, there are more male patients (43%) than normally in GO; it might be due to the known association of a higher severity of GO with the male gender (Oeverhaus et al., 2023). Furthermore, our patients were much older (almost 10 years) than a usual GO study cohort, which might also reflect the association of older age with more severe GO, especially concerning motility (Oeverhaus et al., 2024). Prior treatments varied, with most patients being post decompression (60%). Orbital decompression, especially of the medial wall, is known to lead to a dislocation of orbital tissues into the ethmoidal sinuses. This results in new development or deterioration of pre‐existing esotropia and often in large angle deviations (Oeverhaus et al., 2019; Paridaens et al., 1998). The fact that not all patients underwent decompression and that eight patients underwent prior orbital irradiation might confound the results.

### 4.2 Surgical success

Almost all large angle deviations could be drastically reduced by tendon elongation with Tutopatch®. This surgical success is in line with various studies reporting the efficacy of the elongation technique (Esser et al., 2011; Oeverhaus, Fischer, Hirche, et al., 2018; Oeverhaus, Fischer, Schlüter, et al., 2018; Wipf et al., 2018). Additional procedures were in a few cases the resection of the opposite muscle as maximal recession/elongation was reached.

Previously, hang‐back procedures were advocated, however, with the disadvantages of using non‐absorbable sutures, and potential scleral mis‐attachment. In a rat study, a hang‐backed muscle was found to move anteriorly in 56% of the cases (Wysenbeek et al., 2004). In patients operated with Tutopatch®, the muscle tendon does not attach posteriorly to the equator of the eyeball (Wipf et al., 2018). We confirm this observation, as this ‘posterior fixation’ effect would have resulted in a limitation of motion towards the operated muscle, which was not seen in our patient series. We found that the duction range remains stable and even tends to increase in case of the horizontal duction range after elongation of the medial rectus muscle with a maximum of 13 mm elongation. In contrast, in a similar study where medial rectus muscle elongation was performed up to 22 mm, a greater reduction in adduction was found (Oeverhaus, Fischer, Hirche, et al., 2018; Oeverhaus, Fischer, Schlüter, et al., 2018). This raises the question if there is a cut‐off point for optimal elongation. We are aware of the fact that successful strabismus surgery depends on many factors, including measurement preoperatively, the surgical strategy, variations in orbital anatomy, orbital fat present and the operative procedure itself (Schutte et al., 2009). In the protocol of this study, we described the procedures in as much detail as possible (see Method section), the surgical strategy was jointly drawn up and the surgery was performed by experts in the field. This does not alter the fact that the outcomes could be negatively influenced by above‐mentioned factors.

Previous studies of elongation surgery are retrospective in nature (Esser et al., 2011; Oeverhaus, Fischer, Hirche, et al., 2018; Oeverhaus, Fischer, Schlüter, et al., 2018; Prinz et al., 2022; Thorisdottir et al., 2018, 2019; Wipf et al., 2018). Thorisdottir et al. (2019) describe his results of 42 patients with GO operated on with donor sclera with a 12% reoperation rate and no adverse effects. However, the material is not commercially available. Prinz et al. (2022) operated on 13 patients with fascia lata on the inferior rectus muscle and found a dose–response of 2.6 ± 2.9 Δ/mm which is lower compared to our dose–response (3.47 ± 1.11 Δ/mm [range 1.64–5.33 Δ/mm]). It is unclear if the material used causes this difference. Our results are comparable with the Tutopatch elongation study of Esser et al. (2011), of 2°/mm (3.5 Δ/mm). No statistical difference was found in dose effect response 6 months after surgery. The inferior rectus elongation showed the highest SD, confirming that the surgical plan of this muscle should be made with extra attention. Regarding all different types of material, no conclusion can be made upon preference except on the availability of material.

Our dose–response found for elongation of the medial rectus muscle (2.48 ± 0.69 Δ/mm) is quite similar compared to the study of Wipf et al. (2018), who followed five patients after bilateral medial rectus tendon elongation for 1–10 years and found at final follow‐up a dose–response of 2.95Δ/mm for distance fixation. A much lower dose–response was found by Oeverhaus, Fischer, Hirche, et al. (2018); Oeverhaus, Fischer, Schlüter, et al. (2018), who operated on a group of 87 patients and found, depending on a unilateral or bilateral procedure, approximal 1.0°/mm (2Δ/mm) effect. However, their total elongation ranged from 12 to 44 mm, in which the force of the muscle decreased significantly.

No dose–response for elongation of the superior rectus is available in literature, but when comparing our data (2.50 ± 0.63 Δ/mm [range 2.00–3.33 Δ/mm]) with the literature of conventional superior rectus surgery in GO patients (3.2 Δ/mm; Eckstein et al., 2021), the dose response is only slightly lower. In a recent study of Huelin et al. (2024) about surgical effects of adjustable sutures in GO patients, dose–responses were also similar (MR 2.37 PD/mm and IR 3.75 PD/mm) dose effect responses presented as in the present study. However, Huelin et al. experienced an overcorrection in 18% of patients, which we did not encounter in our series of patients with large elongation.

The dose–response of cyclotorsion was clinically particularly significant for lengthening the inferior rectus (primary position +0.86 ± 0.26°/mm and downgaze +0.71 ± 0.55°/mm) and for the effect of the elongation of the rectus superior in downgaze (−0.68 ± 0.68°/mm). The latter can be explained by improved depression limitation in abduction, which is often seen when the rectus superior is enlarged, reducing the overaction of the obliquus superior of the other eye.

Of interest, lengthening of the inferior rectus did not provoke incyclotorsion in down gaze, contrary to the conventional large muscle recession (Jellema et al., 2012, 2023). With the elongation technique, depression remains preserved and even increases the total duction range. As a result, based on Hering's law, the superior obliques are not triggered to overact. It might be that the superior rectus and superior oblique in our case series were not enlarged as mentioned in the literature as an argument for intorsion after strabismus surgery (Del Porto et al., 2019; Wei et al., 2016). Unfortunately, we neither incorporate MRI findings, nor did we measure depression in abduction.

### 4.3 Quality of life

Although 11 patients (31%) needed additional surgery, we found that already after the elongation surgery that functional GO‐QoL improved significantly by an average of 31 points. This is in line with previous prospective studies of conventional strabismus surgery QoL in GO patients (Jellema et al., 2014, 2017). It may be that patients, due to being aware of participating in the study, completed the questionnaires differently (Hawthome effect). The time and place of answering the questions may also influence the outcome. Our study did not include a control group without intervention. Therefore, we cannot rule out that the observed improvement in patient reported outcomes was to some extent due to regression to the mean.

To the best of our knowledge, this is the first study to include the HADS questionnaire in strabismus surgery. The effect size was moderate, 0.627. However, although overall HADS level decreased significantly (preoperatively 11.5 to postoperatively 5.7 points), both values were below the clinically relevant cut‐off score of 15 points.

The HADS questionnaire has previously been administered to a Chinese group of consecutive euthyroid GO patients in a study of Wong and Yu (2013), where they found a cut‐off threshold of 10/11 for the entire questionnaire and, respectively 4/5 and 6/7, for depression and anxiety. Compared with our study, the value for strabismus surgery is similar (11.5 points), but much lower (5.7 points) after treatment. However, the patient groups in both studies are not comparable. In Wong's study, consecutive patients were asked to participate, and it is unknown which ophthalmic treatment they underwent. In addition, cultural differences may play a role.

### 4.4 Limitations

This study also has limitations, other than the before mentioned confounders of surgical success rates, performance bias (Hawthorne effect) and regression to the mean. The different participating centres are located in three countries, which means that cultural differences can influence the QoL (Lee & Sundar, 2020). Although measurement criteria were set both pre‐ and postoperatively, observer bias cannot be ruled out. Related to this, the study methods allowed freedom of choice in the surgical plan. This may have led to a higher rate of additional muscle surgery. In addition, we did not allow any use of prism glasses during the investigation, which can lead to a smaller field of BSV and underrating the outcome of surgery. The current results can be helpful as a guideline for future research.

## 5 CONCLUSION

In conclusion, the current study shows that muscle tendon elongation is efficient in patients with severe strabismus, with the advantage of not weakening the action of the elongated muscle. In 69% of this severely impaired patient group, a single intervention is sufficient to create a useful field of binocular single vision and subsequently improve QoL and anxiety and depression significantly within 3 months after surgery.
